# Sarcoma of the breast: breast cancer history as etiologic and prognostic factor—A population-based case–control study

**DOI:** 10.1007/s10549-020-05802-3

**Published:** 2020-07-21

**Authors:** Fredrik Karlsson, Fredrik Granath, Karin E. Smedby, Jan Zedenius, Robert Bränström, Inga-Lena Nilsson

**Affiliations:** 1 Department of Molecular Medicine and Surgery, Karolinska Institutet, Stockholm, Sweden; 2 Department of Breast, Endocrine Tumours and Sarcoma, Karolinska University Hospital, Stockholm, Sweden; 3 Department of Medicine Solna, Division of Clinical Epidemiology, Karolinska Institutet, Stockholm, Sweden

**Keywords:** Breast sarcoma, Angiosarcoma, Radiation induced, Incidence, Survival

## Abstract

**Purpose:**

Sarcomas of the breast account for about 1% of all breast malignancies. The aim of this national survey was to explore etiologic and prognostic factors.

**Methods:**

Utilizing national Swedish registers, all patients registered with mesenchymal tumors in the breast during the period 1993–2013 (*n* = 344) were identified and compared to up to ten age and gender matched controls. Cancer history was retrieved for cases and controls. Conditional Poisson regression models were used for calculation of odds ratios.

**Results:**

Previous breast cancer was overrepresented among patients with angiosarcoma. The highest risk occurred ≥ 5 years after treatment for breast cancer (OR 73.9, 95% confidence interval, CI, 25.4–215; *P* < 0.001). An increase in incidence of angiosarcoma was observed during the study period (1.10, 95% CI 1.05–1.16; *P* < 0.001). The overall incidence of breast sarcoma increased from 1.52 to 2.04 cases per million per year. Angiosarcoma of the breast was associated with a significant excess mortality compared to age-matched controls (HR 4.65, 95% CI 3.01–7.19; *P* < 0.001).

**Conclusions:**

Angiosarcoma increased in incidence and displayed a more severe clinical course, with significantly shorter survival. The strong association between a history of breast cancer 5 years or more prior to the diagnosis of angiosarcoma points to radiotherapy as a contributing factor.

**Electronic supplementary material:**

The online version of this article (10.1007/s10549-020-05802-3) contains supplementary material, which is available to authorized users.

## Introduction

Sarcomas are rare neoplasms arising from mesenchymal cells. There are various types of sarcomas, classified by morphology and cell-type of origin, and the classification systems are still evolving [1–3]. The tumors can originate from any part of the body. In general, soft tissue sarcoma is reported to represent about 1% of all malignant tumors in adults [4]. Despite a large number of epidemiological studies of the different types of sarcomas, the reported incidence varies greatly [5–13]. This may in part be explained by underreporting, but also by problems identifying cases from cancer registries. There is no uniform coding system for sarcomas, which makes identification of cases difficult. Most registers are based on ICD coding, which is topographic rather than morphological. The true incidence for sarcoma thus remains unclear. This also makes prognostic studies more difficult to perform and interpret. The present study is an attempt to address these issues by exploring the nationwide Swedish registers.

Breast sarcomas, i.e., breast tumors derived from mesenchymal cells, account for about 1% of all breast malignancies and consist of several different histopathological subtypes. The current understanding of the etiology and prognosis of these tumors is still limited. The most common histopathological subtypes are angiosarcoma and malignant phyllodes tumors [14, 15]. Phyllodes tumors are composed of both epithelial cells and connective tissue stroma and range from benign (grade 1) to overtly malignant (grade 3). The malignant phyllodes tumors have a higher proportion of mesenchymal proliferation and have a genetic profile similar to other breast sarcoma, but not to angiosarcoma [16]. Angiosarcoma is a subtype of sarcoma originating from blood- or lymphatic vessels. Previous irradiation is an established risk factor for its development [17]. Causal links to individual susceptibility dependent on gene-environment interactions have been suggested [18, 19]. Chronic lymphoedema after a mastectomy with lymph node dissection together with irradiation is another reported risk factor for lymphangiosarcoma (Stewart Treves syndrome) [20]. The aims of this national survey were to analyze the incidence of breast sarcoma during a 20-year period, to evaluate risk in relation to previous malignancies and to assess prognosis in the same individuals.

## Materials and methods

The study was performed as a matched case–control study. A database with gathered information from multiple data sources was used to identify cases and controls. The database includes information from the Swedish Cancer Register, the Swedish Cause-of-Death register and the Swedish Population register. The Swedish Cancer Register is mandatory for all malignant diagnoses and contains data from 1958 [21]. It includes data on social security number, sex, place of residency at diagnosis, reporting hospital and clinic, date of diagnosis, clinical and morphological diagnosis, and extent of tumor at diagnosis. The completeness of the Swedish Cancer Register has been estimated to be > 96% [22]. The Swedish Cause-of-Death Register contains data from 1961 and includes data on time and place of death and the cause coded according to ICD classification. The Swedish Population Register contains data on all Swedish citizens and migration in and out of the country. The patient database also contains information from national censuses including socio-economic data such as educational level. All these registers have the social security number as a common parameter and can therefore easily be cross referenced.

A combination of site-coding according to ICD 7 (170, corresponding to ICD 10 C50.X) and morphological coding was used to find cases of mesenchymal tumors/sarcomas of the breast during the period 1993 to 2013. A total of 344 cases were identified (Table 1). Each case was matched according to sex and age to up to ten controls from the general population. Angiosarcoma and lymphangiosarcoma were grouped as angiosarcoma. Phyllodes tumors were divided in three sub-classes according to histopathology (benign, borderline or malignant). Due to relatively small numbers the benign phyllodes tumors, that are not mandatory to register in the cancer registry, were omitted from further analyses. The most uncommon sarcomas were grouped as “other sarcoma.” A proportion of these were not specified with histopathological coding according to SNOMED and were classified as sarcoma not otherwise specified (NOS). For patients treated in the Stockholm county, information of previous breast cancer treatment was collected from the patients’ medical records.

**Table 1:** Distribution of breast sarcoma

Breast sarcoma	Histology	SNOMED	*n*	Age at index
Subgroups				Median (min–max)
Angiosarcoma	Hemangiosarcoma	91203	44	70 (26–91)
	Lymphangiosarcoma	91703	2	62 (61–63)
Phyllodes	malignant	90203	177	58 (18–89)
	borderline	90201	67	52(17–84)
Other sarcoma	Sarcoma NOS^#^	88003	24	69 (19–98)
	Spindle cell sarcoma	88013	6	71 (48–92)
	Giant cell sarcoma	88023	1	60
	Fibrosarcoma	88103	5	72 (58–89)
	Aggressive fibromatosis	88211	1	35
	Malignant fibrous histocytoma	88303	3	82 (74–88)
	Liposarcoma	88503	3	62 (52–72)
	Leiomyosarcoma	88903	7	62 (18–98)
	Endometrial stromal sarcoma	89303	2	54 (52–55)
	Osteosarcoma	91803	1	77
	Malignant peripheral nerve sheat tumor	95403	1	32

Except for one male diagnosed with a liposarcoma, all patients were females

^#^Not otherwise specified

### Statistical analysis

Statistics were calculated with SPSS v26 and SAS 9.4. Incidence was calculated as number of identified cases per year divided by the total population in Sweden, male and female, at the end of the same year. A Poisson regression model was used for comparison of incidence trends over time. Survival analyses were performed by the Kaplan–Meier method and Cox regression analysis. By study design, for survival analyses, the controls assigned to each case were censored at the time for death of the case. Conditional regression models were used for calculation of odds ratios.

## Results

**Fig. 1:**
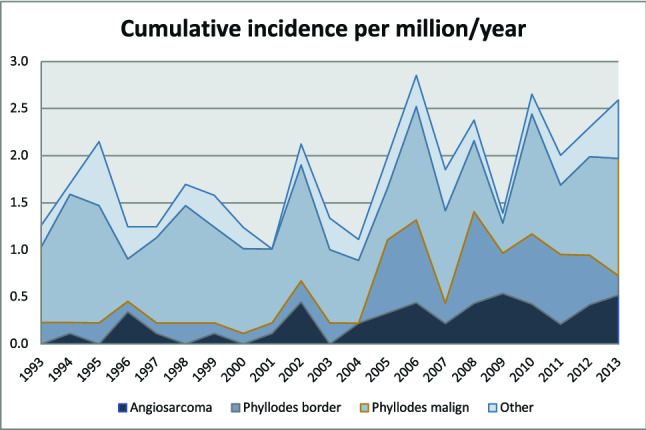
Cumulative incidence of breast sarcomas, including borderline and malignant phyllodes tumours

During the period 1993–2013, 344 patients were registered with mesenchymal tumors in the breast (Table 1). Patients with phyllodes tumors were younger (median age 56.3 (range 17–89) than patients with angiosarcoma (median age 70.5 (range 26–91), *P* < 0.001). The study period was divided into four groups encompassing 5–6 calendar years each (Table 2). The incidence of angiosarcoma increased from 0.09 during the first period 1993–1998 to 0.42 in 2009–2013 (trend of increase in the incidence: 1.10, 95% CI 1.05–1.16; *P* < 0.001). The overall incidence of breast sarcoma increased from a mean incidence of 1.52 cases per million per year during the first half of the study period to 2.04 during the second period. (Fig. 1).

**Table 2:** Relative risk per time period adjusted for gender and age

Breast sarcoma subgroups	Year interval	*n*	Relative risk	95% CI	*P*^*#*^	Incidence per 1,000,000	95% CI incidence
Angiosarcoma	1993–1998	5	1.00			0.09	0.03–0.22
	1999–2003	6	1.45	0.44–4.75	0.540	0.14	0.05–0.30
	2004–2008	15	3.59	1.30–9.88	0.013	0.34	0.19–0.56
	2009–2013	20	4.47	1.68–11.9	0.003	0.42	0.26–0.65
Phyllodes, malignant	1993–1998	53	1.00			1.00	0.75–1.31
	1999–2003	42	0.91	0.61–1.37	0.667	0.92	0.66–1.24
	2004–2008	38	0.80	0.53–1.22	0.300	0.80	0.57–1.10
	2009–2013	44	0.90	0.60–1.34	0.602	0.90	0.65–1.21
Phyllodes, borderline	1993–1998	9	1.00			0.17	0.78–1.35
	1999–2003	7	0.98	0.34–2.49	0.878	0.16	0.75–1.36
	2004–2008	26	3.34	1.56–7.13	0.002	0.57	0.69–1.28
	2009–2013	25	3.03	1.42–6.50	0.004	0.52	0.57–1.10
Other sarcoma	1993–1998	15	1.00			0.28	0.16–0.47
	1999–2003	10	0.78	0.35–1.74	0.550	0.22	0.11–0.41
	2004–2008	14	1.05	0.51–2.18	0.895	0.30	0.16–0.50
	2009–2013	15	1.04	0.51–2.14	0.905	0.30	0.17–0.49

The incidence is standardized for age and sex

^#^vs reference

### History of breast cancer

Thirty-three out of 46 patients diagnosed with angiosarcoma in the breast had a history of breast cancer; 25 were ipsilateral, two were contralateral and data on laterality was missing in six cases. The risk of angiosarcoma of the breast was strongly associated with a history of breast cancer (Table 3). The highest risk occurred ≥ 5 years after the first breast cancer with a peak 5–10 years after, OR 167, CI 95% 35.1–791; *P* < 0.001. Also, for sarcoma other than phyllodes, an increased risk was observed 5 years or more after treatment for breast cancer (HR 6.58, 95% CI 2.13–20.3; *P* < 0.001). In the borderline phyllodes group there were no cases with history of cancer less than 5 years before diagnosis. No increased risk of development of sarcoma in the breast after having malignant tumors other than breast cancer was observed (Table 3).

**Table 3:** Breast sarcoma, odds ratio (OR) in relation to earlier cancer history

Breast sarcoma subgroups	Breast cancer						Other cancer					
	0–5 yrs			> 5 yrs			0–5 yrs			> 5 yrs		
	OR	95% CI	*P*	OR	95% CI	*P*	OR	95% CI	*P*	OR	95% CI	*P*
Angiosarcoma	1.28	0.20–81.2	0.907	73.9	25.4–215	< 0.001	0.55	0.09–32.4	0.774	2.6	0.49–13.7	0.262
Phyllodes, malignant	0.90	0.21–3.88	0.887	0.93	0.28–3.08	0.911	1.87	0.62–5.66	0.267	1.23	0.55–2.76	0.615
Phyllodes, borderline	0.00	0.00–n/a	0.985	0.77	0.10–6.09	0.805	0.00	0.00–n/a	0.608	1.27	0.29–5.54	0.754
Other sarcoma	2.73	0.57–13.0	0.208	6.58	2.13–20.3	0.001	1.01	0.11–9.38	0.992	0.76	0.18–3.33	0.719

Information on previous breast cancer and treatment were available for angiosarcoma patients and control persons treated at Stockholm County only (Supplementary Table). All had received radiation therapy in doses between 42 and 50 Gy. One of the patients developed angiosarcoma on the contralateral side. This patient had a history of postoperative radiotherapy and a locoregional recurrence on the thoracic wall, which was considered inoperable but was stable on hormone therapy. The angiosarcoma in the upper lateral quadrant of the contralateral breast was diagnosed 17 years after the postoperative radiotherapy and might be considered to be a so called primary angiosarcoma. All patients in the control group with history of breast cancer in whom we could obtain previous medical records had received radiotherapy in similar doses (data not shown).

### Survival analyses

**Fig. 2:**
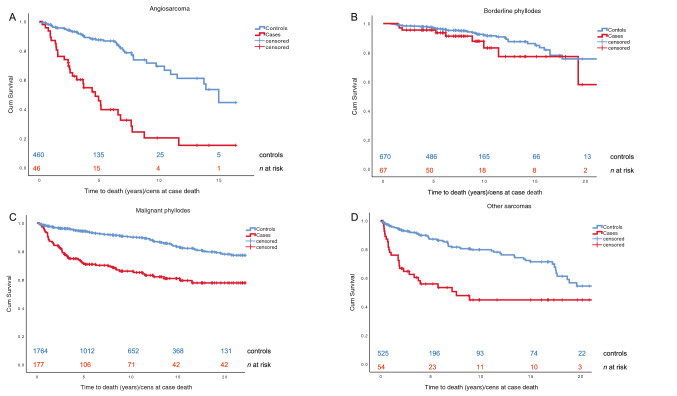
Kaplan-Meier survival plots comparing cases and controls for the different sarcoma diagnoses in the study

Median follow-up time was 7.6 years (range 0–23). Overall survival was analyzed for the separate histopathologic groups compared with controls. (Fig. 2a–d) The occurrence of a breast sarcoma was associated with a significantly shorter survival. Median survival time in the angiosarcoma group was 4.4 years (95% CI 3.13–6.17) and for unspecified sarcoma 5.7 years (95% CI 0–11.6). For the other categories, median time could not be calculated or was not reached during follow-up. Compared with the controls, increased mortality rates were observed during the first 5 to 7 years after diagnosis for patients registered with angiosarcoma and malignant phyllodes (*p* < 0.001). For patients with borderline phyllodes tumors (*n* = 67) survival did not differ from controls.

Survival did not differ significantly between sarcoma patients with and without previous breast cancer, but a history of other cancer than breast cancer was a significant adverse prognostic factor with OR 1.95 (95% CI 1.12–3.40). No difference related to educational level was observed (data not shown).

Survival data were stable over time and did not differ between the first and second half of the time period under study.

## Discussion

The main findings of this study were the strong association between the risk of angiosarcoma and a history of ipsilateral breast carcinoma, not observed for the other histopathological subtypes, and the prognostic similarities between malignant phyllodes tumors and breast sarcoma. Angiosarcoma, often seemingly triggered by radiotherapy following breast cancer surgery, displayed the most severe clinical course, with significantly shorter survival.

Radiotherapy has been utilized in cancer treatment since its discovery in the nineteenth century. In the 1970s, breast conservative surgery in combination with postoperative radiotherapy was introduced. During the following decades, utilization of this treatment method increased and it is now dominant [21, 23].

Breast sarcoma contributes to a very small percentage of all breast malignancies. Angiosarcoma, the most common sarcoma subtype, has been reported to account for only 0.1–0.2% of all malignant breast neoplasms [24]. The reported incidences of angiosarcoma following breast cancer treatment varies between 0.01 and 1% [25, 26]. When comparing incidence figures between studies, the population at risk is obviously a key factor. In this study, the entire population of Sweden has been covered. In contrast, although male breast cancer might be underreported [22], these tumors almost exclusively affect women. If the population at risk would be considered to be only the female population, this would double the incidence.

We observed an increasing incidence of angiosarcoma over time and we suspect that this, at least in part, may be explained by radiation as a part of breast cancer treatment. There was also a slight increase in incidence of borderline phyllodes that may be explained by a shift in diagnosis, since the reported cases of benign phyllodes tumors decreased during the same period. When the reported benign phyllodes tumors were included in analysis we observed a stable overall incidence during the study period (data not shown).

The risk of breast sarcoma was strongly associated with a history of breast cancer, displaying a peak incidence 5–10 years after the diagnosis of breast cancer. The fact that a majority of angiosarcomas (73%) occurred on the same side as the previous breast cancer is consistent with several reports of sarcomas developing in previously irradiated fields [18, 27–30].

Individual data on previous radiotherapy was available for a subset of the patients, all of whom had received radiation in doses of 42–50 Gy. We assume that a majority of the angiosarcoma patients with previous breast carcinoma may have received radiotherapy and that irradiation was a contributing factor for subsequent angiosarcoma development. It is also possible that the risk association between other sarcomas 5–10 years after the diagnosis of breast cancer could be explained by inclusion of some misclassified angiosarcomas in this group. Radiation-induced breast sarcoma was recently reported to be a clinicopathologically distinct type of radiation-induced malignancy with significantly shorter latency from time of initial radiation, different recurrence patterns and potentially better outcomes after radical surgical treatment compared to other radiation-induced sarcomas [31].

Radiation-induced breast angiosarcoma has also been associated with a worse prognosis than primary angiosarcoma [32–34]. Based on a recent survey from Italy, including 112 patients treated at 24 centers, only 50.5% of the patients were still alive after 5 years follow-up, and the disease-free survival was only 35 months [32]. In our cohort, no such difference was obvious, possibly due to the limited size of the cohort. Previous breast cancer per se did not affect differences in survival between the groups and neither was there any difference in survival observed between the early and the late period of the study.

The prognosis for breast sarcoma patients is poor. We observed increased mortality among patients with angiosarcoma and malignant phyllodes during the first 5 to 7 years after diagnosis compared to controls. On the contrary, borderline phyllodes tumors did not lead to shortened overall survival. Conditional regression analysis showed a weak correlation between age at diagnosis and survival time.

Concern has been raised that the incidence of radiation-associated angiosarcoma will increase in the future due to extended use of radiation therapy in treatment of breast cancer including in situ cancer. Our study is consistent with this assumption. In terms of absolute or relative risk of developing angiosarcoma after radiation to the breast, this study can not provide an exact quantification, since it is not designed to include patients at risk (all patients receiving radiotherapy). Since angiosarcoma is still very rare, we can assume that the absolute risk for an individual receiving radiotherapy is low. However, we observed a fourfold increase in the incidence of angiosarcoma in the later study period from 0.09 to 0.42, which calls for concern about future incidence of this tumor type. These findings are in accordance with recently published data from a Finnish national survey [35].

In order to study rare diagnoses and outcomes, a large study population is mandatory. The strength of this study is the completeness of the Swedish national registers, which cover the entire population and are well validated. The population analyzed is relatively large. However, studies of rare tumors are always a challenge. We have to consider risk of underreporting and the fact that the coding practice of sarcomas is not fully standardized. When investigating rare diseases such as sarcomas, each case that is misdiagnosed or missed in registration will affect the overall incidence.

## Conclusion

Previous ipsilateral breast carcinoma was a strong risk factor for development of breast sarcoma. The observed trend of increase in the incidence of angiosarcoma during the study period could hypothetically be related to changed routines regarding the use of irradiation following breast cancer treatment, and further studies on this connection are of great clinical importance.

## Electronic supplementary material

Below is the link to the electronic supplementary material.

Supplementary file1 (PDF 59 kb) Supplementary table. Patient data for a subset of angiosarcoma patients and controls with history of breast carcinoma.
