# Supplementary material for: Sarcoma of the breast: breast cancer history as etiologic and prognostic factor—A population-based case–control study
Source: Breast Cancer Res Treat. 2020 Jul 21;183(3):669–75. doi: 10.1007/s10549-020-05802-3 (PMC7497680; doi:10.1007/s10549-020-05802-3)
Supplement: Supplementary file 1 — Supplementary file1 (PDF 59 kb) Supplementary table. Patient data for a subset of angiosarcoma patients and controls with history of breast carcinoma. [file 10549_2020_5802_MOESM1_ESM.pdf]

Age	Time since BC	Side	Surgery for BC	Histo BC	Chemo	Hormone therapy	RT (Gy)	Surgery for AS	Histo AS	Misc
**80**	10	ipsi	partial ME+ axillary nodes	9 mm ductal carcinoma. ER+, PR+, no LN met	none	Tamoxifene	42	ME	37 mm AS Ki-67 80%	MRM contralat 9 years previous, DCIS
**84**	7	ipsi	partial ME+SNB	17mm ductal carcinoma. Elston I. ER+, PR+, Her2-, SNB no met	none	Tamoxifene	45.22	ME +LD	22 mm mutlifocal AS. Grade II	Subcutaneous metastases 2 years post surgery for AS
**85**	9	ipsi	partial ME+SNB	10 and 14 mm lobular carcinoma. Elston II. ER+, PR+, Ki-67 12%	none	Arimidex	50	ME + axillary nodes	60 mm AS. No LN met.	
**69**	17	contra	partial ME, completed MRM axillary nodes	40 mm poorly diff lobular carcinoma and LCIS. Met in 1/6 LN	none	Anastrozol	46	ME + axillary nodes	40 mm AS. Grade III. No LN met.	
**77**	5	ipsi	partial ME+SNB	10 mm ductal carcinoma. Elston II. ER+, PR+ , SNB - (0/5 LN)	none	Tamoxifene	42 ipsi, 42.56 contra	ME+LD	20 mm AS. Grade II	3 years after first cancer contralateral ductal carcinoma 10 mm. Partial ME +SNB, no met, postop RT

Supplementary table, Data on previous breast carcinoma for a subset of angiosarcoma patients
